# β-Hydroxyisovaleryl-Shikonin Exerts an Antitumor Effect on Pancreatic Cancer Through the PI3K/AKT Signaling Pathway

**DOI:** 10.3389/fonc.2022.904258

**Published:** 2022-07-04

**Authors:** Yu Zeng, Haifeng Zhang, Minghui Zhu, Qingfan Pu, Jinhai Li, Xiao Hu

**Affiliations:** Department of Hepatobiliary Surgery, Ruian People’s Hospital, The Third Affiliated Hospital of Wenzhou Medical University, Wenzhou, China

**Keywords:** pancreatic cancer, β-hydroxyisovaleryl-shikonin, apoptosis, antitumor effect, PI3K/AKT

## Abstract

Pancreatic cancer (PC) is marked with a low survival rate and lack of recognized effective treatment strategy. We investigated the antitumor effect of β-hydroxyisovaleryl-shikonin (β-HIVS) on PC and the associated working mechanism. Cell toxicity was determined using Cell Counting Kit-8 (CCK-8) assay. Acridine Orange/Ethidium Bromide (AO/EB) double-fluorescent staining assay accompanied by flow cytometry was utilized to estimate cell apoptosis. Cell cycle, reactive oxygen species (ROS), and mitochondrial membrane potential were all evaluated using flow cytometry. Transwell and wound healing assays were performed to evaluate cell migration and invasion. Protein expression was analyzed by Western blots. A xenograft mouse model was employed to determine the antitumor effect of β-HIVS *in vivo.* PC cell viability gradually decreased with increasing β-HIVS while apoptosis was enhanced together with cell-cycle blockage in the G0–G1 phases. β-HIVS induced mitochondrial dysfunction, ROS production, and DNA damage and inhibited the invasive and migratory ability of PC cells. We further confirmed the suppression of EMT and PI3K/AKT pathways as underlying mechanisms. The mouse model treated with the increasing dose of β-HIVS displayed decreased tumor growth rate, along with increased apoptosis. Thus, β-HIVS exerts antitumor effects on PC through inducing apoptosis, ROS production, decreasing mitochondrial membrane potential, and suppressing signal pathways, such as PI3K/AKT. In summary, β-HIVS might be a promising strategy for PC treatment.

## Introduction

Pancreatic cancer (PC) is a lethal carcinoma with high mortality. Its mortality rate is disproportionately higher, relative to its incidence (1–3). The early diagnosis of PC is quite difficult, leading to belated treatment. As expected under such circumstances, PC patients frequently suffer from poor prognosis (1). For most PC patients, surgery is not possible because of end-stage disease and, therefore, conventional chemotherapy remains the major treatment option (4). However, conventional chemotherapy has its own limitations. Thus, there is an urgent need to establish effective treatment strategies by identifying novel therapeutic targets for PC.

Shikonin, a naphthoquinone compound, is isolated from the traditional Chinese medicine, named, comfrey. Its potential applications have been reported for a variety of diseases/conditions, such as cancer, inflammation, and wound healing (5). The antitumor effects of shikonin involve enhanced cytotoxicity, cell apoptosis, reduced tumor cell invasion and migration, and tumor angiogenesis (6). β-Hydroxyisovaleryl-shikonin (β-HIVS) is a naturally occurring syringin product, and its *in vitro* growth inhibitory and apoptosis-inducing effects on leukemia (7), cervical cancer (8), and lung cancer cell lines (9) has been demonstrated. However, any potential effect of β-HIVS on PC cells has never been determined. One study suggested a possible role of reactive oxygen species (ROS) in β-HIVS’s anticancer activity against the human leukemia cell line, HL60 cell line, and human lung carcinoma, DMS114 cell line (10).

ROS has great significance and diverse roles in regulating many physio-pathological processes, including signal transduction, cell proliferation, cell survival, promoted oxidative damage, and cell death. However, how ROS affects tumor therapy is still controversial (11). For example, a study (12) found that decreased deoxycytidine kinase can enhance drug resistance to gemcitabine by increasing ROS levels. In the meantime, several natural products have been shown to induce apoptosis and autophagy through ROS production and cell-cycle arrest (13, 14).

We explored the antitumor efficiency of β-HIVS on PC cells and interpreted their functional mechanism. The findings in the study may help identify advanced therapeutic targets against PC.

## Materials and Methods

### Cell Culture

The pancreatic adenocarcinoma cell line, PANC-1, was obtained from CAS. Cells were cultured in RPMI-1640 medium, supplemented with 10% heat-inactivated fetal calf serum (Gibco, Grand Island, NY, USA). Besides these, 2 mM L-glutamine and 100 U/ml antibiotics like P/S were added into the culture medium (Gibco, USA). Cells were maintained in moist air containing 5% CO_2_ at 37°C.

### CCK-8 Assay

The cell viability of PANC-1 cells when treated with various concentrations (0, 1, 2.5, 5, 10, 20, 40, 80, 160, and 320 μM) of β-HIVS was detected using CCK-8 assay. One hundred microliters of medium containing 3,000 cells per well was placed into 96-well plates, and cells were allowed to adhere. Then, cells were treated with indicated concentrations of β-HIVS for 24 h. Each well containing 10% CCK8 was incubated at 37°C for another 2 h. The absorbance data at 450 nm (OD450) were determined using a microplate reader. All the experiments were performed three times, and the data are shown as mean ± S.E.

### Cell Apoptosis

PC cells were divided into different experimental groups, as indicated. Firstly, AO/EB double-fluorescent staining assays were conducted to evaluate the apoptotic PANC-1 cells among the groups. Cells (0.6 × 10^6^/well) were seeded into six-well plates. After treatment, cells were soaked into 70% ethanol for 20 min for fixation and then washed with phosphate-buffered saline (PBS). Thereafter, 1 μg AO and 1 μg EB were used for staining in each well for 10 min, followed by capturing of the pre-stained cells using a fluorescent microscope (BD Biosciences, Franklin Lakes, NJ, USA). Secondly, another apoptosis assay was performed. Cells were harvested after treatment as mentioned above and then resuspended using an Annexin V and PI mixture solution (Beyotime, Shanghai, China). The mixture was incubated for 15 min at room temperature with protection from light. Finally, cells were examined by a flow cytometer (Beckman Coulter, Brea, CA, USA) to identify the rate of apoptotic cells. All the experiments were performed three times, and the results are displayed as mean ± S.E. for each group.

### Cell-Cycle Assay

Cells in all four groups were incubated in six-well plates, with 2 × 10^5^ cells in each well, overnight following a 2-h treatment. Thereafter, the collected cells were resuspended in cold PBS with PBS discarded afterward. Seventy percent of cold ethanol was utilized to fix the collected cells at 4°C for more than 8 h. Cells were subjected to incubation with 100 μg/ml PI solution for 30 min at 4°C protecting from light. At last, the cell cycle was detected using the flow cytometer, while distribution was analyzed using FlowJo software. All the experiments were performed three times.

### ROS Detection and Mitochondrial Membrane Potential Assay

Cellular ROS content and mitochondrial membrane potential (MMP) level were determined using 2′,7′-dichlorofluorescein diacetate (DCFH-DA, Beyotime, China) staining and mitochondrial cationic fluorescent dye Rhodamine 1023 (Sigma-Aldrich, St. Louis, MO, USA), respectively. To detect the ROS, we measured the fluorescence intensity of the stained cells using a flow cytometer with the following parameters: excitation at 480 nm and emission at 525 nm. Besides that, 488 and 530 nm as excitation and emission wavelengths were applied for the MMP assay. N-Acetyl-cysteine (NAC) was used as a positive control to consume ROS in the experiment. Cells were then split into the following four groups: (1) PANC-1 cells without treatment (Control group); (2) 20 μM β-HIVS-treated cells (20 μM group); (3) cells co-treated with 20 μM β-HIVS and 10 μM NAC (20 μM β-HIVS + NAC group); and (4) 10 μM NAC alone-treated cells (NAC group). All the experiments were performed three times.

### Transwell Assays

Cell culture plates inserted with Transwell chambers were used. The Transwell chambers with 8-μm pore size (Corning, Tewksbury, MA, USA) were utilized for invasion and migration assay. The number of invaded PANC-1 cells was determined using Matrigel (BD Biosciences, USA) coating, while the cell migratory ability was assessed using transwell without Matrigel coating. The upper and lower chambers had RPMI-1640 medium supplemented with different ratios of FBS, 0.1% and 20% for the upper and lower chambers, respectively. Cells were cultured for 48 h and then fixed using 4% PFA for 30 min. Thereafter, the chamber was washed in PBS and water alternately and then finally soaked in crystal violet solution for 30 min to stain the cells. The cells remaining in the upper chamber were scrubbed using cotton-tipped swabs, while the cells that crossed from the upper chamber to the lower chamber were imaged using an electronic microscope (Olympus, Tokyo, Japan). ImageJ was used to count the stained cells. Three independent experiments were performed, and the results are displayed as mean ± S.E.

### Wound Healing Assay

Wound healing assay was conducted in a six-well plate. Cells were routinely cultured as monolayers, and a 2-mm scratch was made along the midline of the plate. Then, the plate was washed to discard the cell debris and remove the old medium. Wells were replaced with drugs dissolved in fresh serum-free medium. The wounding area was measured after 24 h, and pictures were captured for the wounding area using a microscope. Five views were captured in each wound randomly. Three independent experiments were performed.

### Western Blotting

Cells in different groups were washed first. Then the culture medium was removed and cells were washed. RIPA buffer was used to extract the proteins (Sigma-Aldrich, USA). The BCA kit (Sigma-Aldrich, USA) was used to measure the absolute protein content for each group. Proteins were electrophoresed on 10% SDS-polyacrylamide gels first. Afterward, the bands on the gels were transferred to PVDF membranes (EMD Millipore, Billerica, MA, USA). Five percent of non-fat dried milk was used for blocking. After blocking, the following primary antibodies were diluted into PBST to be used overnight at 4°C—BAX, Bcl-2, Caspase-3, P53, CyclinD1, Cyclin E, β-tubulin, γ-H2AX, E-Cadherin, N-Cadherin, ZO-1, MMP2, MMP9 (MMP here is different from the former one; the former stands for mitochondrial membrane potential, while the latter stands for matrix metalloproteinases), PI3K, and GAPDH. An MMP9 primary antibody was diluted 2,000 times while other primary antibodies were all diluted 1,000 times. GAPDH was used as an internal reference. After incubation with primary antibodies, the membranes were incubated in PBST with non-fat milk containing horseradish peroxidase (HRP)-conjugated secondary antibody (1: 5,000; Sannon, China) for 1 h at RT. The bands on the membrane were developed and recorded using an imager. The intensity of the protein bands was quantified through ImageJ software.

### Xenograft Tumor Model

Twenty-four female nude mice aged 6 weeks were housed. First of all, we prepared 100 µl PBS containing 5 × 10^6^ PC cells. Subsequently, these cells were injected into the left back of mice. The cells were injected subcutaneously. The purpose of these two steps was for generating a pancreatic cancer xenograft model. The 24 mice were randomly split into a total of four groups. The four groups were control group (no treatment), 10-mg/kg group (10 mg/kg β-HIVS administered to each mouse), 20-mg/kg group (20 mg/kg β-HIVS administered to each mouse), and 40-mg/kg group (40 mg/kg β-HIVS administered to each mouse). The tumor length and width and the mice"s body weight for every experimental subjective were recorded four times using vernier calipers and a weighing machine. Tumor length and width was used to calculate the final tumor volume:


$$Tumor\left(mm^{3}\right)=\frac{\pi \times length\times width^{2}}{6}$$

Mice were executed after 14 days of treatment and tumors were isolated to record length and weigh.

### TUNEL Assay

TUNEL assay was conducted on tumor slices to record apoptotic cell ratios, following the manufacturer’s (Roche, Basel, Switzerland) instructions. Sliced specimens were fixed with 4% PFA, and the residual PFA was washed using PBS. This was repeated three times. Then 0.2% Triton X-100 was applied to permeabilize cells. After treatment with 50 μl TUNEL working solution, apoptotic cells were detected by excitation at 488 nm, with cells marked by green fluorescence under a fluorescence microscope.

### Statistical Analysis

SPSS 20.0 and GraphPad were utilized to execute the data analysis. Student’s t-test was used to calculate the P value between groups, while comparisons among three groups or more were conducted by one-way ANOVA. A P value less than 0.05 was considered as significantly different. In our presented results, * stands for P < 0.05, ** stands for P < 0.01, and *** stands for P < 0.001.

## Results

### β-HIVS Inhibits PC Cell Proliferation by Promoting Cell Apoptosis

**Figure 1:**
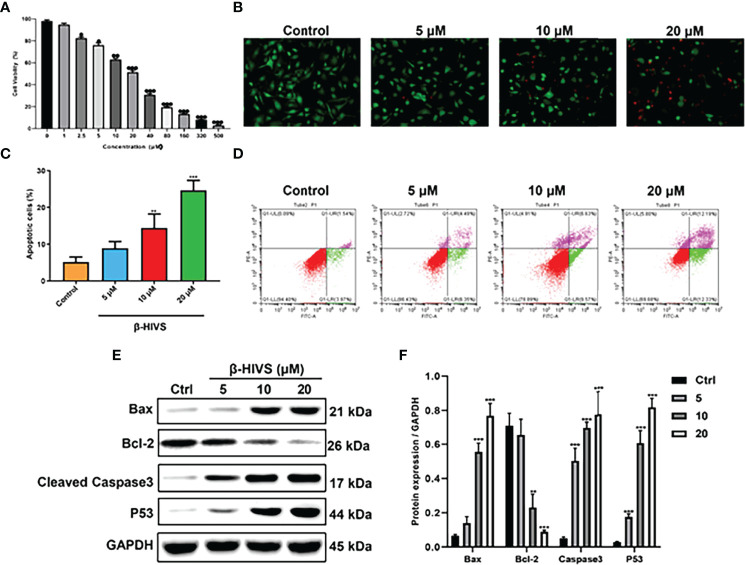
β-HIVS induced PC cell toxicity and apoptosis. **(A)** Cell toxicity of β-HIVS on PANC-1 cells was determined using CCK-8 assay. **(B)** Apoptotic cells detected using AO/EB double staining. **(C)** Apoptotic cells after treatment with the different concentrations of β-HIVS. **(D)** Apoptotic cells after double fluorescence staining using Annexin V-FITC, and PI labeling using flow cytometry. **(E)** Representative Western blot of the apoptosis-related proteins including Bcl-2, BAX, P53, Caspase-3, and GAPDH. **(F)** Corresponding protein expression of **(E)**. **(A)** *P<0.05, **P<0.01, ***P<0.001 compared to the 0μM β-HIVS group; **(C, F)** **P<0.01, ***P<0.001 compared to the Control group.

As depicted in **Figure 1A**, cell proliferation of PANC-1 cells gradually decreased with increasing concentrations of β-HIVS, which means cell toxicity was evident as the concentration of β-HIVS increased from 0 to 500 μM. Based on the above results, we selected cells treated with β-HIVS at the following four concentrations, namely, 0 μM, 5 μM, 10 μM, and 20 μM, for the subsequent experiments. As seen in the AO/EB double staining (**Figure 1B**), a decreasing number of green cells and an increasing number of red cells were detected when β-HIVS concentrations were increased. These observations suggested that β-HIVS promotes the apoptosis of PC cells. Strikingly, flow cytometry analysis supported these conclusions and provided further evidence that β-HIVS could promote PC cell apoptosis. Particularly, the 10-μM group * or 20-μM group ** significantly increased the apoptotic cells, compared to the group without treatment (**Figures 1C, D**). Moreover, the apoptosis-related protein levels, such as, Bcl-2, BAX, P53, and Caspase-3, in PANC-1 cells were significantly changed after β-HIVS treatment with the results supporting apoptosis induction (P < 0.001; **Figures 1E, F**).

### β-HIVS Arrests the PC Cell Cycle in the G0/G1 Phase

**Figure 2:**
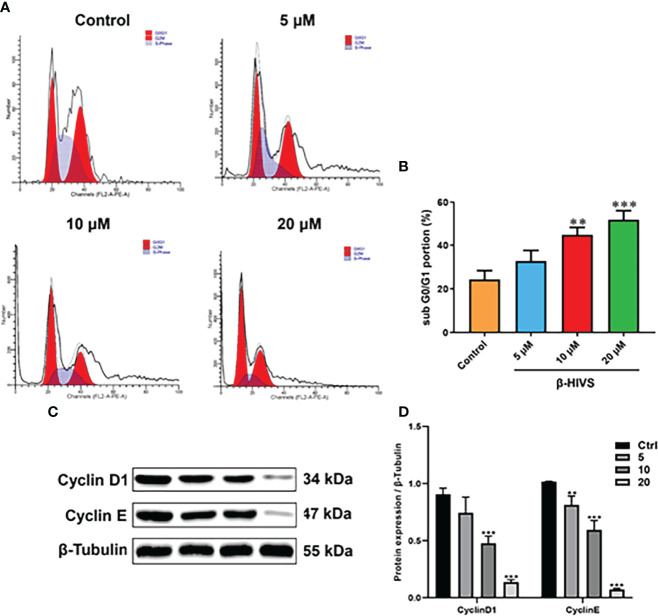
How β-HIVS affects cell-cycle progression in PC. **(A)** Cell-cycle distributions detected using flow cytometry. **(B)** Cells arrested in the G0/G1 phase. **(C)** Representative Western blots exhibiting the expression of proteins including Cyclin-D1, Cyclin-E, and β-tubulin. **(D)** Relative protein quantified level of bands from Western blots. **(B, D)** **P<0.01, ***P<0.001 compared to the Control group.

According to our flow cytometry results, β-HIVS arrests PANC-1 cells in the G0/G1 phase (**Figure 2A**). Notably, more cells were found in the G0/G1 phase when β-HIVS was added, particularly its increasing concentrations. When treated using 10 μM β-HIVS, PANC-1 cells in the G0/G1 phase** were significantly increased; even more, G0/G1 phase cells were observed when cells were treated with 20 μM β-HIVS (**, **Figure 2B**). Moreover, the expression of Cyclin D1 and Cyclin E decreased continuously with the increasing concentration of β-HIVS (**Figures 2C, D**). In summary, based on the presented results, we could demonstrate that β-HIVS has the ability to affect PC cell-cycle progression.

### β-HIVS Induces Mitochondrial Dysfunction, ROS Overproduction, and DNA Damage

**Figure 3:**
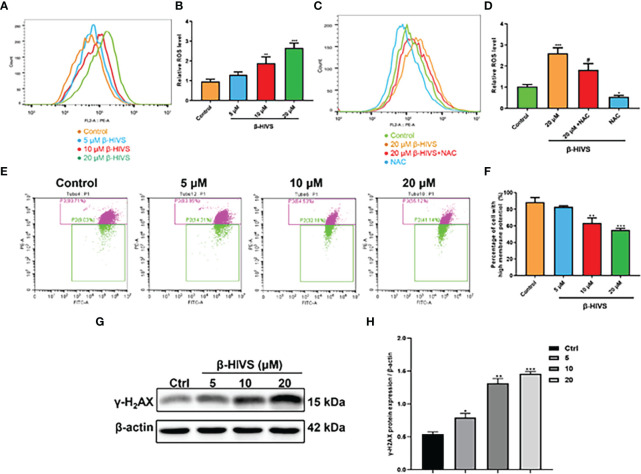
Mechanisms underlying the cell toxicity of β-HIVS on PC cells. **(A)** ROS levels in cells treated with variety of concentrations of β-HIVS observed using flow cytometry. **(B)** Relative ROS levels in cells quantified for the observations from flow cytometry in **(A)**. **(C)** ROS level response to β-HIVS or NAC measured using flow cytometry. **(D)** Relative ROS levels in **(C)**. **(E)** MMP levels in cells treated withβ-HIVS with concentration variation detected using flow cytometry. **(F)** Relative MMP levels in **(E, G)** Western blot analysis of γ-H2AX expression. **(H)** Relative protein expression of γ-H2AX quantified from **(G)**. **(B, D, F, H)** *P<0.05, **P<0.01, ***P<0.001 compared to the Control group; ^#^P<0.05 compared to the 20μM β-HIVS group.

In accordance with the flow cytometry results, ROS levels in PANC-1 cells were significantly enhanced when the concentrations of β-HIVS increased (**Figures 3A, B**) with a dose-dependent increase, while NAC could eliminate the effect of β-HIVS on ROS production in the cells (*, **Figures 3C, D**). Further, with the increasing concentrations of β-HIVS, the MMP of PANC-1 cells decreased significantly. Specifically, cells with high MMP decreased, among which the ratio reduced from 89.71% to 55.12% (P < 0.001) (**Figures 3E, F**). Furthermore, we found that the expression of a DNA damage marker, γ-H2AX, increased with increasing concentrations of β-HIVS. The expression level was highest when cells were treated with 20 μM β-HIVS (P < 0.001) (**Figures 3G, H**).

### β-HIVS Inhibits PC Cell Invasion and Migration

**Figure 4:**
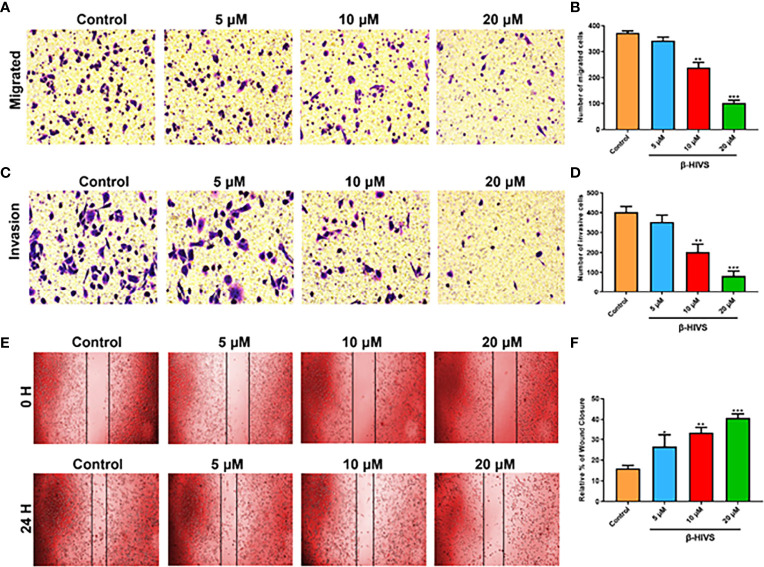
Effect of β-HIVS on PC cell invasion and migration. **(A)** Representative photographs from transwell assays. **(B)** The migrated cells were quantified for each group. **(C)** Images from transwell invasion assays. **(D)** The invasive cells were quantified in each treatment group. **(E)** Images of the scratch-wound healing assay. **(F)** Relative percentages of wound closure. **(B, D, F)** *P<0.05, **P<0.01, ***P<0.001 compared to the Control group.

As is shown in **Figures 4A, B**, as the concentration of β-HIVS increased, fewer cells migrated, as detected using transwell migration assay (P < 0.01, P < 0.001). Similarly, markedly fewer PC cells invaded in response to increasing concentrations of β-HIVS (**Figures 4C, D**). Meanwhile, the scratch-wound healing experiment provided more evidence that β-HIVS inhibited PC cell migration (**Figures 4E, F**). Thus, through a few different experiments, we were able to conclusively observe that β-HIVS inhibits the migration and invasion of PC cells in a dose-dependent manner.

### β-HIVS Represses the EMT Process and PI3K/AKT Pathway in PC Cells

**Figure 5:**
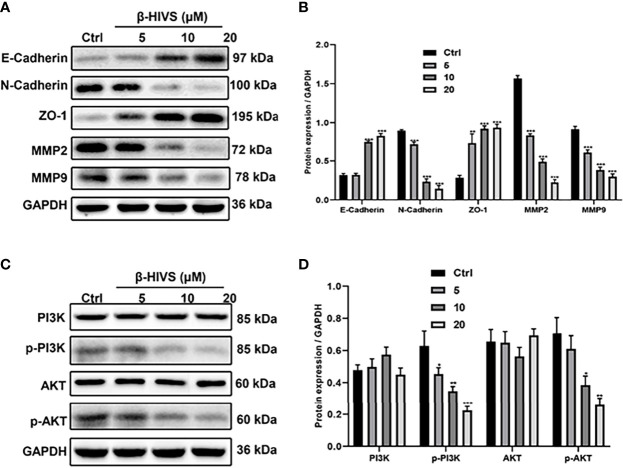
β-HIVS effects on the EMT process and PI3K/AKT pathway in PC cells. **(A)** Representative Western blot images of E-Cadherin, N-Cadherin, ZO-1, MMP2, MMP9, and GAPDH. **(B)** Relative quantification of Western blot bands from **(A)**. **(C)** Representative blot images of PI3K, p-PI3K, AKT, p-AKT, and GAPDH. **(D)** Relative quantification of Western blot bands from **(C)**. **(B, D, F)** *P<0.05, **P<0.01, ***P<0.001 compared to the Control group.

We observed that with increasing concentrations of β-HIVS, more E-Cadherin and ZO-1 proteins were expressed, as determined by Western blot analysis, while markedly less N-Cadherin, MMP2, and MMP9 proteins were expressed simultaneously in PC cells (**Figures 5A, B**), suggesting that β-HIVS can effectively inhibit the EMT process in PC cells. Furthermore, treatment with the increasing concentrations of β-HIVS led to a significant downregulation of p-PI3K and p-AKT, which could reveal that β-HIVS inhibited the PI3K/AKT pathway as well in the PC cells (**Figures 5C, D**).

### β-HIVS Restrains Tumor Growth *In Vivo*

**Figure 6:**
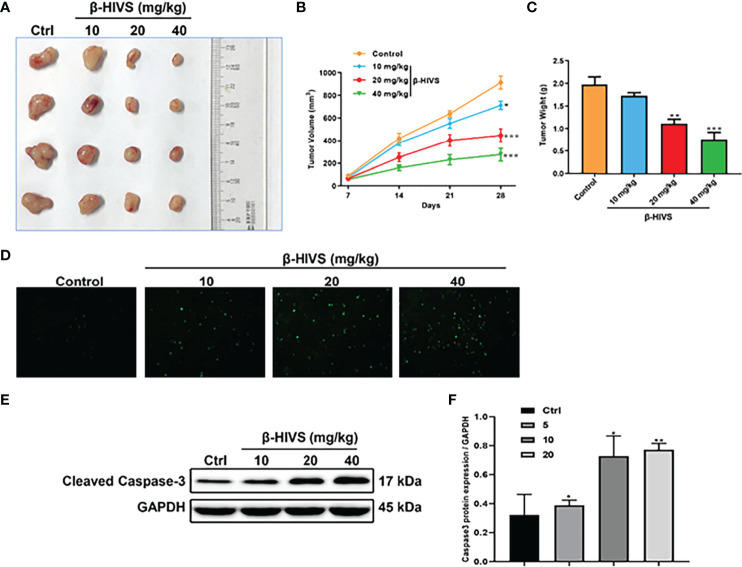
Effect of β-HIVS on the *in vivo* tumor xenograft model. **(A)** Images of xenograft tumors in each treatment group. **(B)** Statistics of tumor volume of mice. **(C)** Statistics of mice’s isolated tumor weight. **(D)** Representative images of TUNEL assay. **(E)** Caspase-3 protein expression detected using Western blot. **(F)** Relative protein expression of Western blots from **(E)**. P<0.05, **P<0.01, ***P<0.001 compared to the Control group.

Based on our observations from the *in vitro* experiments, we further conducted *in vivo* experiments. We found that the increasing dose of β-HIVS inhibited tumor growth in mice (**Figure 6A**). Mice treated with β-HIVS displayed a significantly inhibited tumor growth rate, compared to the control group (**Figures 6B, C**). Meanwhile, more apoptotic cells were detected in isolated tumors, using TUNEL assay (**Figure 6D**). Additionally, we further confirmed that Caspase-3, a pro-apoptotic protein, was upregulated in tumors from the mice that were administered β-HIVS (*, **) (**Figures 6E, F**), thus confirming induction of apoptosis in mice that were administered β-HIVS. In summary, our *in vivo* observations fully supported our *in vitro* observations.

## Discussion

The incidence of PC is predicted to rise worldwide by 2030, with new cases of PC and related deaths more than tripling, while there are still no effective treatment strategies or therapeutic targets for PC (15). In this study, we demonstrate that β-HIVS could act as an effective therapeutic agent for PC. Cell toxicity and more cell apoptosis was detected with the application of β-HIVS *in vitro*. Moreover, the trend was found to be dose-dependent. In addition, the results we got from the *in vivo* experiment were consistent with the *in vitro* experiment. Similarly, Lu et al. (8) found that β-HIVS inhibits cervical cancer cell proliferation and promotes their apoptosis depending on drug dose. Moreover, they also found that only 6% of cervical cancers were in the S phase. Another study (16) found that naphthoquinones could block different melanoma cells mainly in the S or G2/M phase. Specifically, the present study identified that β-HIVS could arrest PC cells in the G0/G1 phase. We also demonstrated that β-HIV could inhibit PC cell invasion and migration *in vitro*. Consistent with these observations, a number of studies (17–19) have found that G0/G1 cell-cycle arrest could prohibit cancer cell migration and invasion.

What is more, multiple studies reported by different groups have revealed an association between the G0/G1 phase blockade and anti-PC effects. Liu et al. (20) found that knockdown of estrogen-related receptor alpha induced apoptosis as well as G0/G1 cell-cycle arrest in PC cells. Wang et al. (21) showed that upregulation of glutathione S-transferase mu-3 significantly inhibited cell proliferation by delaying the G0/G1 phase transition. Xue et al. (22) observed that miR-125b could be downregulated in pancreatic ductal adenocarcinoma. On the contrary, upregulation of miR-125b could arrest the cells in the G0/G1 phase. Notably, the studies we mentioned here have concluded that the underlying mechanisms of PC inhibition involve the PI3K/AKT signaling pathway (22) or ROS accumulation (21). Consistently, we here presented evidence that β-HIVS promotes ROS accumulation and inhibits the PI3K/AKT signaling pathway, while ROS accumulation can be reversed by the ROS scavenger NAC. In addition, we found that mitochondrial dysfunction and DNA damage could be induced by β-HIVS, which further led to antitumor activity.

It has been reported that changes in MMP and ROS are associated with EMT progress (23). Moreover, we have provided evidence for the production of ROS by β-HIVS. This is in line with the production of ROS by several other natural products as well as dietary agents (24–26). Here, we found that PANC-1 cells treated with β-HIVS exhibited a significantly increased level of ROS while MMP and proteins related to EMT process were markedly reduced. Viera et al. (27) observed that the accumulation of ROS causes DNA damage, resulting in an anti-PC effect. We also found that the DNA damage marker γ-H2AX was expressed more when treated with more β-HIVS, which are consistent with the abovementioned findings.

## Conclusion

In conclusion, both *in vitro* and *in vivo* study in this work identified a potential role of β-HIVS in anti-PC treatment. β-HIVS can promote apoptosis and ROS accumulation in PC cells. It also reduces MMP and cell viability. Furthermore, anticancer effects could be realized by intervening the EMT process and signaling pathway, for example, PI3K/AKT pathway. The application of β-HIVS might work as an emerging promising strategy for PC treatment.

## Data Availability Statement

The raw data supporting the conclusions of this manuscript will be made available by the authors, without undue reservation, to any qualified researcher upon request.

## Ethics Statement

The animal study was reviewed and approved by the Ruian People’s Hospital Animal Ethics Committee.

## Author Contributions

YZ and XH conceived the project. YZ and HZ performed the experiments. YZ, HZ, and MZ extracted and analyzed the data. QP and JL performed the statistical analysis and drafted the paper. YZ and XH revised the manuscript. All authors contributed to the article and approved the submitted version.

## Funding

This study was financially supported by the Ruian Science and Technology Foundation (MS2020014).

## Conflict of Interest

The authors declare that the research was conducted in the absence of any commercial or financial relationships that could be construed as a potential conflict of interest.

## Publisher’s Note

All claims expressed in this article are solely those of the authors and do not necessarily represent those of their affiliated organizations, or those of the publisher, the editors and the reviewers. Any product that may be evaluated in this article, or claim that may be made by its manufacturer, is not guaranteed or endorsed by the publisher.
